# Management of patients with locally recurrent rectal cancer with a previous history of distant metastases: retrospective cohort study

**DOI:** 10.1093/bjsopen/zrae061

**Published:** 2024-06-13

**Authors:** Luca Sorrentino, Elena Daveri, Filiberto Belli, Raffaella Vigorito, Luigi Battaglia, Giovanna Sabella, Filippo Patti, Giovanni Randon, Filippo Pietrantonio, Claudio Vernieri, Davide Scaramuzza, Sergio Villa, Massimo Milione, Alessandro Gronchi, Maurizio Cosimelli, Marcello Guaglio

**Affiliations:** Colorectal Surgery Unit, Fondazione IRCCS Istituto Nazionale dei Tumori, Milan, Italy; Translational Immunology Unit, Fondazione IRCCS Istituto Nazionale dei Tumori, Milan, Italy; Colorectal Surgery Unit, Fondazione IRCCS Istituto Nazionale dei Tumori, Milan, Italy; Department of Radiology, Fondazione IRCCS Istituto Nazionale dei Tumori, Milan, Italy; Colorectal Surgery Unit, Fondazione IRCCS Istituto Nazionale dei Tumori, Milan, Italy; First Pathology Division, Fondazione IRCCS Istituto Nazionale dei Tumori, Milan, Italy; Radiation Oncology Unit, Fondazione IRCCS Istituto Nazionale dei Tumori, Milan, Italy; Department of Medical Oncology, Fondazione IRCCS Istituto Nazionale dei Tumori, Milan, Italy; Department of Medical Oncology, Fondazione IRCCS Istituto Nazionale dei Tumori, Milan, Italy; Department of Medical Oncology, Fondazione IRCCS Istituto Nazionale dei Tumori, Milan, Italy; Translational Immunology Unit, Fondazione IRCCS Istituto Nazionale dei Tumori, Milan, Italy; Radiation Oncology Unit, Fondazione IRCCS Istituto Nazionale dei Tumori, Milan, Italy; First Pathology Division, Fondazione IRCCS Istituto Nazionale dei Tumori, Milan, Italy; Sarcoma Surgery Unit, Fondazione IRCCS Istituto Nazionale dei Tumori, Milan, Italy; Colorectal Surgery Unit, Fondazione IRCCS Istituto Nazionale dei Tumori, Milan, Italy; Colorectal Surgery Unit, Fondazione IRCCS Istituto Nazionale dei Tumori, Milan, Italy

## Introduction

The management of locally recurrent rectal cancer (LRRC) is challenging, requiring both complex surgery and a multidisciplinary approach to optimize the chance of cure^1–3^. About 30–50% of instances of LRRC occur with distant metastases and, traditionally, a history of previous or current metastatic disease has often been considered a contraindication for curative treatment^4,5^. However, there is limited evidence on the impact of distant metastases on LRRC outcomes^5–7^. The improvements in outcomes for metastatic colorectal cancer^8,9^ are changing the paradigm of treatment for LRRC with distant metastases and question the previous approach of best supportive care in this patient group.

The aim of the present study was to assess the impact of previous or synchronous distant metastases on 3-year disease-free survival (DFS) and 3-year overall survival (OS) rates for patients with LRRC treated with curative intent.

## Methods

### Study population

All consecutive patients affected by LRRC who were treated at the Colorectal Surgery Unit of the National Cancer Institute of Milan (Italy) from January 2009 to September 2022 were selected from a prospectively maintained database. The database was locked in September 2023 to allow a minimum follow-up of 12 months. Authorization for the present study by the Institutional Review Board was obtained (‘SEBASTIAN’ project, protocol no. 149/2019). Based on pelvic MRI, patients with LRRC were grouped according to the classification developed at the National Cancer Institute of Milan^10^.

### Study design and endpoints

All patients initially treated with curative intent were included in the analyses. Patients were divided into three groups: those with a previous history of distant metastatic disease, synchronous or metachronous to primary rectal cancer (M+ with primary rectal cancer); those with metastases synchronous to local relapse (LRRC with M+); and those without any previous or current history of metastases (LRRC without M+). Survival outcomes of M+ with primary rectal cancer *versus* LRRC with M+ *versus* LRRC without M+ were assessed by univariable and multivariable analyses. The primary endpoints of the study were the 3-year DFS and 3-year OS rates. Secondary endpoints were the 3-year re-local recurrence-free survival and 3-year distant progression-free survival rates for the three groups. A sub-analysis was then performed including only patients who completed the planned multimodal therapy with curative resection (or local treatment) of both LRRC and distant metastases. Details regarding the multimodal treatment for LRRC and statistical analyses are available in the *Supplementary Methods*.

## Results

### Study cohort

Of 3584 patients with primary rectal cancer, LRRC occurred in 203 patients (5.7%) in the primary institution. A further 46 patients who were treated for their primary tumour in other hospitals were treated for LRRC in the primary institution. Of the 249 patients with LRRC, 15 patients (6.0%) had M+ with primary rectal cancer, 24 patients (9.7%) had LRRC with M+, and 210 patients (84.3%) had LRRC without M+. A total of 182 patients completed the planned multimodal treatment (*Fig. S1*). The baseline characteristics of primary rectal cancer and LLRC are available in *Tables S1*, *S2*. Details regarding the treatment of metastatic disease are available in *Table S3*.

### Molecular features of locally recurrent rectal cancer

*KRAS* was mutated in 34.8% of LRRC patients with known *KRAS* status, with no differences observed between groups (*P* = 0.561). *MSI-H* tumours accounted for 2.8% of LRRC patients with known microsatellite status, with no differences observed between groups (*P* = 0.504). All patients with LRRC with available molecular data were negative for *HER2* amplification and *BRAF* mutation. Next-generation sequencing (NGS) results from surgical specimens or biopsies were available for 14 LRRC patients. The genes that were most frequently mutated were *TP53* (85.7%), *APC* (57.1%), and *KRAS* (50.0%), as can be seen in the complete NGS panel (*Fig. S2*).

### Survival analyses for all included patients and specifically for patients who completed the planned multimodal treatment

**Fig. 1:**
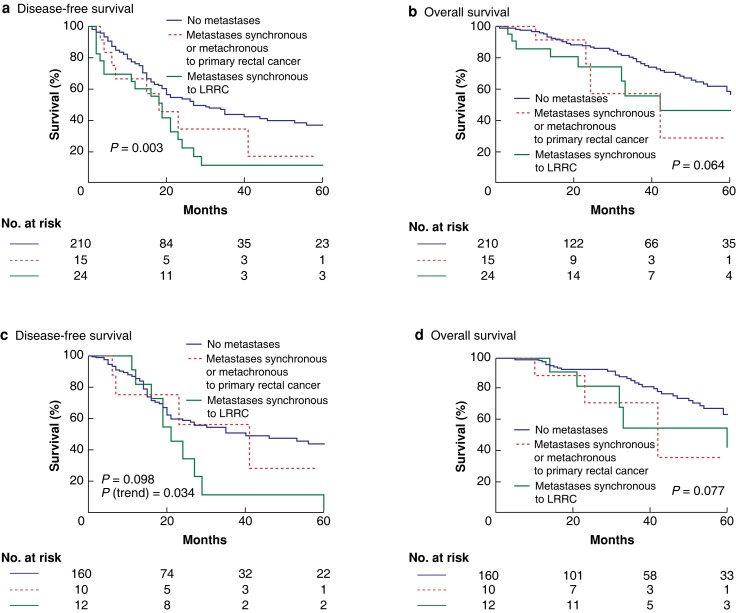
Survival

The median follow-up was 32.6 (range 6–152) months. LRRC without M+ patients had a 3-year DFS rate of 43.8%, whereas M+ with primary rectal cancer patients had a 3-year DFS rate of 34.3%. LRRC with M+ patients had a 3-year DFS rate of 11.1% (log rank *P* = 0.003) (*Fig. 1a*). The 3-year OS rates were 77.5%, 57.3%, and 56.0% respectively (log rank *P* = 0.064) (*Fig. 1b*). When the same outcomes were analysed considering only the 182 patients who completed the planned multimodal treatment, no differences were observed between groups. Indeed, the 3-year DFS rates were 56.3% for M+ with primary rectal cancer patients, 11.4% for LRRC with M+ patients, and 50.6% for LRRC without M+ patients (log rank *P* = 0.098, *P* for trend = 0.034) (*Fig. 1c*). The 3-year OS rates were 71.1%, 54.5%, and 81.5% respectively (log rank *P* = 0.077) (*Fig. 1d*). Analyses of secondary endpoints are available in the *Supplementary Results* (*Fig. S3*).

**a** Disease-free survival for patients with previous metastases and primary rectal cancer, for patients with locally recurrent rectal cancer with concurrent distant metastases, and for patients with locally recurrent rectal cancer without concurrent distant metastases. **b** Overall survival for patients with previous metastases and primary rectal cancer, for patients with locally recurrent rectal cancer with concurrent distant metastases, and for patients with locally recurrent rectal cancer without concurrent distant metastases. **c** Disease-free survival specifically for patients who completed the planned multimodal treatment with curative intent. **d** Overall survival specifically for patients who completed the planned multimodal treatment with curative intent. LRRC, locally recurrent rectal cancer.

### Predictors of worse disease-free survival for LRRC patients

The only independent predictors of worse DFS that emerged from multivariable Cox analyses were R+ surgery for LRRC (HR 3.07, 95% c.i. 1.87 to 5.10; *P* < 0.001) and distant metastases synchronous to LRRC (HR 1.72, 95% c.i. 0.99 to 2.86; *P* = 0.044). LRRC resection with curative intent was independently associated with improved DFS, especially in the case of extended colorectal re-excision (HR 0.38, 95%c.i. 0.19–0.71, *P* = 0.003). The results of the Cox analyses are available in *Table 1*.

**Table 1:** Univariable and multivariable Cox regression analyses for disease-free survival

	Univariable		Multivariable	
	HR (95% c.i.)	*P*	HR (95% c.i.)	*P*
Distance from the anal verge	1.00 (0.96, 1.03)	0.958	–	–
**Multimodal therapy for primary tumour**				
Chemoradiation	2.11 (1.35, 3.41)	0.002	1.06 (0.52, 2.13)	0.880
Chemo only	2.23 (1.31, 3.82)	0.003	1.74 (0.77, 3.95)	0.186
None	Reference	Reference	Reference	Reference
**(y)pT stage of primary tumour**				
ypT0–2	Reference	Reference	Reference	Reference
ypT3	1.82 (1.19, 2.86)	0.007	1.31 (0.69, 2.57)	0.426
ypT4	2.15 (1.21, 3.76)	0.008	1.35 (0.61, 2.96)	0.46
**(y)pN stage of primary tumour**				
ypN0	Reference	Reference	Reference	Reference
ypN1	1.95 (1.17, 3.16)	0.008	1.15 (0.71, 1.90)	0.569
ypN2	1.94 (1.30, 2.88)	0.001	1.66 (0.91, 2.97)	0.091
**Localization of LRRC**				
S1a–b	Reference	Reference	Reference	Reference
S1c	1.29 (0.79, 2.11)	0.311	0.73 (0.39, 1.32)	0.298
S2	1.38 (0.77, 2.41)	0.262	0.55 (0.26, 1.13)	0.109
S3	1.77 (1.12, 2.83)	0.016	0.71 (0.41, 1.25)	0.238
**Multivisceral involvement**				
Yes	1.10 (0.69, 1.68)	0.671	–	–
No	Reference	Reference	–	–
**Lateral pelvic sidewall involvement**				
Yes	1.38 (0.94, 2.00)	0.091	–	–
No	Reference	Reference	–	–
**Margin status of LRRC**				
R0	Reference	Reference	Reference	Reference
R+	3.65 (2.52, 5.36)	<0.001	3.07 (1.87, 5.10)	<0.001
**Type of surgery**				
Rectal re-excision	0.32 (0.21, 0.48)	<0.001	0.50 (0.29, 0.85)	0.012
Extended rectal re-excision	0.33 (0.19, 0.57)	<0.001	0.38 (0.19, 0.71)	0.003
Partial/total exenteration	0.45 (0.20, 0.90)	0.037	0.49 (0.19, 1.13)	0.109
Re-excision with sacrectomy	0.26 (0.09, 0.61)	0.005	1.13 (0.34, 3.30)	0.831
No resection/others	Reference	Reference	Reference	Reference
**Timing of metastatic occurrence**				
Synchronous or metachronous to primary	1.92 (0.94, 3.50)	0.051	1.29 (0.57, 2.61)	0.512
Synchronous to LRRC	2.21 (1.34, 3.48)	0.001	1.72 (0.99, 2.86)	0.044
No metastases	Reference	Reference	Reference	Reference
**Metastasis dominant location**				
Liver	1.25 (0.49, 3.37)	0.645	–	–
Lung	0.82 (0.33, 2.13)	0.67	–	–
Others	Reference	Reference	–	–
**Peritoneal metastases**				
Yes	1.11 (0.48, 2.35)	0.801	–	–
No	Reference	Reference	–	–

LRRC, locally recurrent rectal cancer.

## Discussion

Two recent studies demonstrated that 5.6–6.4% of rectal cancer patients experience local recurrence during their follow-up and up to 41–44.9% of these patients have metastases^11,12^. Excluding patients with known distant metastases, approximately 30% of patients with LRRC have indeterminate lung nodules observed at diagnosis^13^. Despite this clinical issue, there is limited evidence about the outcomes of LRRC patients with distant metastases^5,7^.

The present study demonstrates that patients with LRRC with a previous history of distant metastases can be considered for treatment with curative intent. The timing of the identification of distant metastases impacts on survival, as M+ with primary rectal cancer patients had improved survival compared with LRRC with M+ patients, with 3-year DFS rates of 34.3% and 11.1% respectively (log rank *P* = 0.003). Interestingly, when outcomes were analysed only for patients who completed the planned multimodal treatment with curative intent, a difference in the 3-year DFS rate was no longer observed between groups (log rank *P* = 0.098). These findings suggest that even distant metastases concurrent to LRRC could have a negligible impact on survival, when all metastatic sites are treated with a multimodal approach. Patients who had LRRC with M+ had a higher rate of R+ pelvic surgery and a higher frequency of lateral localization. Treatment with curative intent in this cohort is more challenging^14,15^. A recent paper from the PelvEx Collaborative demonstrated that combined surgery is feasible, with a 30-day mortality rate of 1.6% and major post-operative complications in 32% of patients; however, only primary rectal cancer was included^16^.

The present study has some limitations, including its retrospective nature and the relatively small sample size. Furthermore, only 15.7% of included LRRC patients had a current or previous history of distant metastases, suggesting a selection bias since only patients in whom multimodal treatment was established with curative intent were included.

In conclusion, a previous history of metastatic disease does not represent an absolute contraindication to multimodal treatment with curative intent in LRRC patients. Conversely, patients with distant lesions occurring synchronous to LRRC should be carefully selected for any attempt of curative treatment.

## Supplementary Material

zrae061_Supplementary_Data

## Data Availability

Data will be available upon request to the corresponding author.
