# Supplementary material for: Management of patients with locally recurrent rectal cancer with a previous history of distant metastases: retrospective cohort study
Source: BJS Open. 2024 Jun 13;8(3):zrae061. doi: 10.1093/bjsopen/zrae061 (PMC11170501; doi:10.1093/bjsopen/zrae061)

**Management of Locally Recurrent Rectal Cancer with previous history of distant metastases: retrospective cohort study**

Luca Sorrentino^a^*, MD, Elena Daveri^b^, PhD, Filiberto Belli^a^*, MD, Raffaella Vigorito^c^, MD, Luigi Battaglia^a^, MD, Giovanna Sabella^d^, MD, Filippo Patti^e^, MD, Giovanni Randon^f^, MD, Filippo Pietrantonio^f^, MD, Claudio Vernieri^f^, MD, PhD, Davide Scaramuzza^b^, MD, Sergio Villa^e^, MD, Massimo Milione^d^, MD, Alessandro Gronchi^g^, MD, Maurizio Cosimelli^a^, MD, Marcello Guaglio^a^, MD

^a^Colorectal Surgery Unit, Fondazione IRCCS Istituto Nazionale dei Tumori, Milan, Italy

^b^Translational Immunology Unit, Fondazione IRCCS Istituto Nazionale dei Tumori, Milan, Italy

^c^Department of Radiology, Fondazione IRCCS Istituto Nazionale dei Tumori, Milan, Italy

^d^1st Pathology Division, Fondazione IRCCS Istituto Nazionale dei Tumori, Milan, Italy

^e^Radiation Oncology Unit, Fondazione IRCCS Istituto Nazionale dei Tumori, Milan, Italy

^f^Department of Medical Oncology, Fondazione IRCCS Istituto Nazionale dei Tumori, Milan, Italy

^g^Sarcoma Surgery Unit, Fondazione IRCCS Istituto Nazionale dei Tumori, Milan, Italy

**Supplementary Materials - Index**

| **Supplementary Methods** |  |
| --- | --- |
| Study population | *pag. 3* |
| Multimodal treatment | *pag. 3* |
| Statistical analyses | *pag. 4* |

| **Supplementary Results** |  |
| --- | --- |
| Study cohort | *pag. 5* |
| Baseline characteristics of primary rectal cancers | *pag. 5* |
| Clinical and pathological features of locally recurrent rectal cancers | *pag. 5* |
| Treatment of metastatic disease | *pag. 5* |
| Analysis of secondary endpoints | *pag. 6* |
| Findings on univariate analysis for disease-free survival | *pag. 6* |

| **Supplementary Figures and Tables** |  |
| --- | --- |
| Supplementary Table S1 | *pag. 7* |
| Supplementary Table S2 | *pag. 8* |
| Supplementary Table S3 | *pag. 10* |
| Supplementary Figure S1 | *pag. 11* |
| Supplementary Figure S2 | *pag. 12* |
| Supplementary Figure S3 | *pag. 13* |
|  |  |

**Supplementary Methods**

*Study population*

Patients were staged by thoraco-abdominal computed tomography (CT) scan, positron emission tomography (PET) and contrast-enhanced pelvic magnetic resonance imaging (MRI) with diffusion-weighted imaging (DWI). According to the classification developed at the National Cancer Institute of Milan, LRRC were classified as anterior (S1) if located axially or anteriorly within the pelvis; posterior (S2), in case of sacral involvement; lateral (S3), in case of lateral pelvic sidewall involvement. Inclusion criteria were: proven diagnosis of LRRC on endoscopic and/or percutaneous biopsy or, alternatively, pelvic lesion with features suggesting malignancy on MRI and/or PET; treatment with curative intent; previous history of rectal cancer. Patients were excluded in case of squamous cell histopathology of primary cancer; previous colon cancer localized beyond 20 cm from the anal verge; treatment with palliative intent.

*Multimodal treatment*

All cases were discussed at the colorectal multidisciplinary tumor board at several steps: MRI-based diagnosis and strategies for biopsy of suspected LRRC, decision on multimodal treatment, planning of surgery based on response to neoadjuvant therapies and finally review of surgical outcomes and evaluation of pathological report. In LRRC patients neoadjuvant (re)chemoradiation with or without induction chemotherapy was proposed according to LRRC resectability evaluated on MRI, patients’ comorbidities, expected radiation-induced toxicities and proximity of small bowel in the pelvic site to be (re)irradiated. The cut-off for considering pelvic re-irradiation was 6 months from treatment of primary rectal cancer. Induction chemotherapy included FOLFOX/XELOX o FOLFIRI/XELIRI with or without anti-VEGF or anti-EGFR based on possible previous administered chemotherapy for primary rectal tumor. Pelvic irradiation consisted in delivery of a total dose of 30-54 Gy with a hyperfractionated scheme, with fractions of 1.2 Gy twice a day or 2 Gy once a day. In radiotherapy-naïve patients, concurrent chemotherapy with 5-fluorouracil or capecitabine was administered. After neoadjuvant treatment, surgical resection of LRRC was planned at the multidisciplinary tumor board. Surgical planning was always shared with the radiologists and surgeons from other specialties, including sarcoma surgery, urology, gynecology and reconstructive surgery. All the procedures were performed by laparotomy. The first step was a thorough abdominal exploration to exclude misdiagnosed diffuse peritoneal metastases. Then, mobilization of the pelvic colon beginning at the sacral promontory was performed to explore the pelvis for definitive confirmation of LRRC resectability, as unexpected intraoperative contraindications to surgery could be found. Intraoperative contraindications for proceeding with surgery were: extensive or multiple infiltrations of the pelvic sidewall or wide involvement of the common or external iliac vessels; sacral involvement above the second vertebra; diffuse peritoneal carcinomatosis. Once confirmed the resectability, dissection was conducted exposing the common and internal iliac vessels and ureters laterally, and along a “beyond-TME” plane posteriorly. In case of sacral invasion, abdominosacral resection was performed. In patients with involvement of anterior genitourinary organs an en bloc multivisceral resection or partial/total exenteration were considered.

In LRRC with M+ patients CT scan was carefully evaluated to assess the distant tumor burden. In case of single-site, oligometastatic disease and resectable pelvic relapse, after induction chemotherapy followed by (re)chemoradiation the priority was given to the resection of LRRC, postponing the possible resection or local treatment of distant metastases in a second procedure, provided that R0 resection was achieved on LRRC. In case of extensive metastatic disease and/or unresectable LRRC, systemic chemotherapy was indicated. Then surgical treatment for LRRC and distant metastases was decided based on clinical response to chemotherapy. In case of partial response/stable disease on distant metastases and partial response on LRRC, pelvic surgery was considered as a first step, possibly followed by metastases resection.

*Statistical analysis*

The rate of R0 resections was reported between M+ with primary rectal cancer, LRRC with M+ and LRRC without M+. Impact of current or previous distant disease vs. no metastases on DFS, OS, RLRFS and DPFS was estimated with the Kaplan-Meier survival method. Categorical variables were compared using a χ2 test or Fisher exact test, while continuous variables were compared using a Student’s T test or non-parametric Wilcoxon test as appropriate. Univariate and multivariate survival analyses for DFS were performed with the Cox proportional hazards regression model. Statistical significance was set at p<0.05 (two-tailed). Data analysis was performed using Prism version 9.0 (GraphPad Software Inc., California, USA).

**Supplementary Results**

*Study cohort*

Distant metastases were predominantly located in lungs (41.0%) and in the liver (35.9%). In 12 cases (30.8%) peritoneal carcinomatosis was observed. From 249 patients who started treatment with curative intent, 16 patients were excluded after induction chemotherapy/(re)chemoradiation due to disease progression on LRRC and/or distant metastases. From the remaining 233 patients, 51 were subsequently excluded due to intraoperative findings of non resectability of LRRC or distant metastases.

*Baseline characteristics of primary rectal cancers*

LRRC patients with previous M+ had more frequently a diagnosis of locally advanced primary rectal cancer, with (y)pN1-2 stage in 84.6% vs. 44.6% of LRRC without M+ (p=0.037), and a trend toward increased (y)pT4 stage was observed in LRRC with M+ compared to the other groups (26.1%, p=0.089). Primary rectal cancers presented with a higher distance from the anal verge (12.9 ±5.6 cm, p=0.002) in patients affected by LRRC with M+. LRRC patients with previous M+ with primary rectal cancer received chemotherapy and/or chemoradiation for primary tumor in 100.0% of cases, conversely to the other groups (p=0.057). All the other variables related to primary rectal cancers are reported in Supplementary Table S1.

*Clinical and pathological features of locally recurrent rectal cancers*

CA19.9 was lower in LRRC without M+ compared to the other groups (28.8 ±105.7 U/mL, p=0.002). LRRC with M+ were characterized by more frequent S3 (lateral) pelvic localization (58.3%, p=0.016), multivisceral involvement (41.7%, p<0.001), higher R+ margins rate after surgery (70.8%, p=0.022), a more frequent administration of multimodal treatment beyond surgery, especially chemotherapy alone (62.5%, p=0.048), and a higher likelihood of intraoperative finding of unresectability (50.0%, p=0.009). All the other variables were reported in Supplementary Table S2.

*Treatment of metastatic disease*

Surgical resection of metastatic disease was performed in 60.0% of patients with distant lesions synchronous or metachronous to primary rectal cancers vs. 25.0% of patients with metastatic lesions synchronous to LRRC, where chemotherapy only (45.8%) or observation without any treatment (12.5%) where more frequently proposed (p=0.174). Timing of metastases treatment, surgical approach and margins status were similar between groups, as reported in Supplementary Table S3.

*Analysis of secondary endpoints*

The 3-yr RLRFS rates were 48.1%, 35.2% and 23.6% (Log-rank p=0.02, Supplementary Fig. S3a), while the 3-yr DPFS rates were 80.3%, 50.3% and 37.5% (Log-rank p<0.0001, Supplementary Fig. S3b). When the same outcomes were analyzed considering only the 182 patients who completed the multimodal treatment with curative intent, no differences were observed for 3-yr RLRFS (Log-rank p=0.263, Supplementary Fig. S3c) and 3-yr DPFS (Log-rank p=0.338, Supplementary Fig. S3d).

*Findings on univariate analysis for disease-free survival*

On univariate Cox analysis, the main predictive features of worse DFS were involved margins at LRRC resection (HR 3.65, 95%CI 2.52-5.36, p<0.0001), distant metastases synchronous to LRRC (HR 2.21, 95%CI 1.34-3.48, p=0.001), administration of chemotherapy (HR 2.23, 95%CI 1.31-3.82, p=0.003) or chemoradiation (HR 2.11, 95%CI 1.35-3.41, p=0.002) for primary rectal cancer, and (y)pT4 stage (HR 2.15, 95%CI 1.21-3.76, p=0.008).

**Supplementary Figures and Tables**

**Supplementary Table S1.** Clinical and pathological features of primary rectal cancers

|  | **LRRC without M+(n=210)** | **M+ with primary rectal cancer (n=15)** | **LRRC with M+ (n=24)** | **P Value** |
| --- | --- | --- | --- | --- |
| **Age (years)** | 60.1 (±12.0) | 60.0 (±11.9) | 59.0 (±12.3) | 0.923 |
| **Gender** |  |  |  | 0.934 |
| Male | 130 (61.9%) | 11 (73.3%) | 16 (66.7%) |  |
| Female | 80 (38.1%) | 4 (26.7%) | 8 (33.3%) |  |
| **Distance from the anal verge (cm)** | 8.0 (±5.5) | 7.0 (±4.7) | 12.9 (±5.6) | 0.002 |
| **Multimodal treatment** |  |  |  | 0.057 |
| Chemoradiation | 110 (52.4%) | 8 (53.3%) | 13 (54.2%) |  |
| Chemotherapy only | 39 (18.6%) | 7 (46.7%) | 7 (29.1%) |  |
| None | 61 (29.0%) | 0 (0.0%) | 4 (16.7%) |  |
| **(y)pT stage** |  |  |  | 0.089 |
| (y)pT0-1-2 | 66 (32.0%) | 0 (0.0%) | 4 (17.4%) |  |
| (y)pT3 | 114 (55.3%) | 11 (78.6%) | 13 (56.5%) |  |
| (y)pT4 | 26 (12.6%) | 3 (21.4%) | 6 (26.1%) |  |
| Not available | 4 | 1 | 1 |  |
| **(y)pN stage** |  |  |  | 0.037 |
| (y)pN0 | 116 (55.4%) | 2 (15.4%) | 9 (39.1%) |  |
| (y)pN1 | 61 (29.9%) | 8 (61.5%) | 10 (43.5%) |  |
| (y)pN2 | 30 (14.7%) | 3 (23.1%) | 4 (17.4%) |  |
| Not available | 6 | 2 | 1 |  |
| **Margins status** |  |  |  | 0.549 |
| R0 | 168 (83.6%) | 15 (100.0%) | 19 (82.6%) |  |
| R+ | 33 (16.4%) | 0 (0.0%) | 4 (17.4%) |  |
| Not available | 9 | 0 | 1 |  |

**Supplementary Table S2.** Clinical and pathological features of LRRC

|  | **LRRC without M+(n=210)** | **M+ with primary rectal cancer (n=15)** | **LRRC with M+ (n=24)** | **P Value** |
| --- | --- | --- | --- | --- |
| **Time to LRRC (months)** | 33.1 (37.8) | 41.5 (±30.0) | 25.9 (±19.1) | 0.416 |
| **CEA (ng/mL)** | 18.1 (70.9) | 43.7 (±88.9) | 14.7 (±21.3) | 0.373 |
| **CA19.9 (U/mL)** | 28.8 (105.7) | 55.4 (±70.5) | 54.9 (±82.5) | 0.002 |
| **Size on MRI (mm)** | 36.4 (23.4) | 43.5 (±25.4) | 48.9 (±52.7) | 0.113 |
| **Localization of LRRC** |  |  |  | 0.016 |
| S1a-b | 71 (33.8%) | 4 (26.7%) | 4 (16.7%) |  |
| S1c | 53 (25.2%) | 2 (13.3%) | 4 (16.7%) |  |
| S2 | 33 (15.7%) | 1 (6.7%) | 2 (13.3%) |  |
| S3 | 53 (25.2%) | 8 (53.3%) | 14 (58.3%) |  |
| **Multivisceral involvement** |  |  |  | <0.001 |
| Yes | 28 (13.3%) | 6 (40.0%) | 10 (41.7%) |  |
| No | 182 (86.7%) | 9 (60.0%) | 14 (58.3%) |  |
| **Vascular involvement** |  |  |  | 0.259 |
| Yes | 17 (8.1%) | 3 (20.0%) | 3 (12.5%) |  |
| No | 193 (91.9%) | 12 (80.0%) | 21 (87.5%) |  |
| **Lateral pelvic sidewall involvement** |  |  |  | 0.013 |
| Yes | 54 (25.7%) | 8 (53.3%) | 11 (45.8%) |  |
| No | 156 (74.3%) | 7 (46.7%) | 13 (54.2%) |  |
| **Positive nodes at LRRC** |  |  |  | 0.041 |
| Yes | 16 (7.6%) | 4 (26.7%) | 3 (12.5%) |  |
| no | 194 (92.4%) | 11 (73.3%) | 21 (87.5%) |  |
| **Margins status LRRC** |  |  |  | 0.022 |
| R0 | 115 (54.8%) | 5 (33.3%) | 7 (29.2%) |  |
| R+ | 95 (45.2%) | 10 (66.7%) | 17 (70.8%) |  |
| **KRAS status** |  |  |  | 0.561 |
| Wild type | 72 (63.2%) | 9 (75.0%) | 11 (73.3%) |  |
| Mutated | 42 (36.8%) | 3 (25.0%) | 4 (26.7%) |  |
| Not available | 96 | 3 | 9 |  |
| **Microsatellite status** |  |  |  | 0.504 |
| MSS | 111 (97.4%) | 12 (100.0%) | 14 (93.3%) |  |
| MSI-H | 3 (2.6%) | 0 (0.0%) | 1 (6.7%) |  |
| Not available | 96 | 3 | 9 |  |
| **Multimodal treatment** |  |  |  | 0.048 |
| (re)chemoradiation | 38 (18.1%) | 3 (20.0%) | 2 (8.3%) |  |
| Chemotherapy + (re)chemoradiation | 63 (30.0%) | 1 (6.7%) | 5 (20.8%) |  |
| Chemotherapy alone | 68 (32.4%) | 8 (53.3%) | 15 (62.5%) |  |
| Immunotherapy | 2 (0.9%) | 0 (0.0%) | 0 (0.0%) |  |
| None (surgery only) | 39 (18.6%) | 3 (20.0%) | 2 (8.3%) |  |
| **Chemotherapy regimen** |  |  |  | 0.175 |
| FOLFOX/XELOX ± anti-VEGF or anti-EGFR | 79 (59.4%) | 2 (22.2%) | 11 (55.0%) |  |
| FOLFIRI/XELIRI ± anti-VEGF or anti-EGFR | 46 (34.6%) | 7 (77.8%) | 9 (45.0%) |  |
| FOLFOXIRI ± anti-VEGF or anti-EGFR | 6 (4.5%) | 0 (0.0%) | 0 (0.0%) |  |
| Pembrolizumab | 2 (1.5%) | 0 (0.0%) | 0 (0.0%) |  |
| **Type of surgery** |  |  |  | 0.009 |
| Rectal re-excision | 114 (54.3%) | 3 (20.0%) | 5 (20.8%) |  |
| Extended rectal re-excision | 25 (11.9%) | 5 (33.3%) | 5 (20.8%) |  |
| Partial/total exenteratio | 10 (4.8%) | 2 (13.3%) | 2 (8.4%) |  |
| Re-excision with sacrectomy | 11 (5.2%) | 0 (0.0%) | 0 (0.0%) |  |
| No resection/others | 50 (23.8%) | 5 (33.3%) | 12 (50.0%) |  |
| **Mets dominant location** |  |  |  | 0.085 |
| Liver | - | 8 (35.9%) | 6 (25.0%) |  |
| Lung | - | 6 (41.0%) | 10 (41.7%) |  |
| Others | - | 1 (23.1%) | 8 (33.3%) |  |
| **Peritoneal mets** |  |  |  | 0.249 |
| Yes | - | 3 (30.8%) | 9 (37.5%) |  |
| No | - | 12 (69.2%) | 15 (62.5%) |  |

**Supplementary Table S3.** Multimodal treatment for metastatic disease

|  | **Synchronous or metachronous to primary rectal cancer**  **(n = 15)** | **Synchronous to LRRC**  **(n = 24)** | **p Value** |
| --- | --- | --- | --- |
| **Treatment of metastatic disease** |  |  | 0.174 |
| Stereotactic radiotherapy | 1 (6.7%) | 1 (4.2%) |  |
| Chemotherapy only | 3 (20.0%) | 11 (45.8%) |  |
| Surgery +/- chemotherapy | 9 (60.0%) | 6 (25.0%) |  |
| No treatment/observation | 0 (0.0%) | 3 (12.5%) |  |
| Not Available | 2 (13.3%) | 3 (12.5%) |  |
| **Timing of surgery on metastases** | **(n = 9)** | **(n = 6)** | 0.766 |
| Metastasis-first approach | 1 (11.1%) | 1 (16.7%) |  |
| Primary tumor/LRRC-first approach | 5 (55.6%) | 4 (66.6%) |  |
| Synchronous treatment | 3 (33.3%) | 1 (16.7%) |  |
| **Surgical approach** |  |  | 1.000 |
| Open surgery | 9 (100.0%) | 6 (100.0%) |  |
| Mini-invasive surgery | 0 (0.0%) | 0 (0.0%) |  |
| **Margins status of metastasectomy** |  |  | 0.756 |
| R0-1 | 8 (88.9%) | 5 (83.3%) |  |
| R2 | 1 (11.1%) | 1 (16.7%) |  |

**Supplementary Fig. S1** Flow-chart of patients selection

**
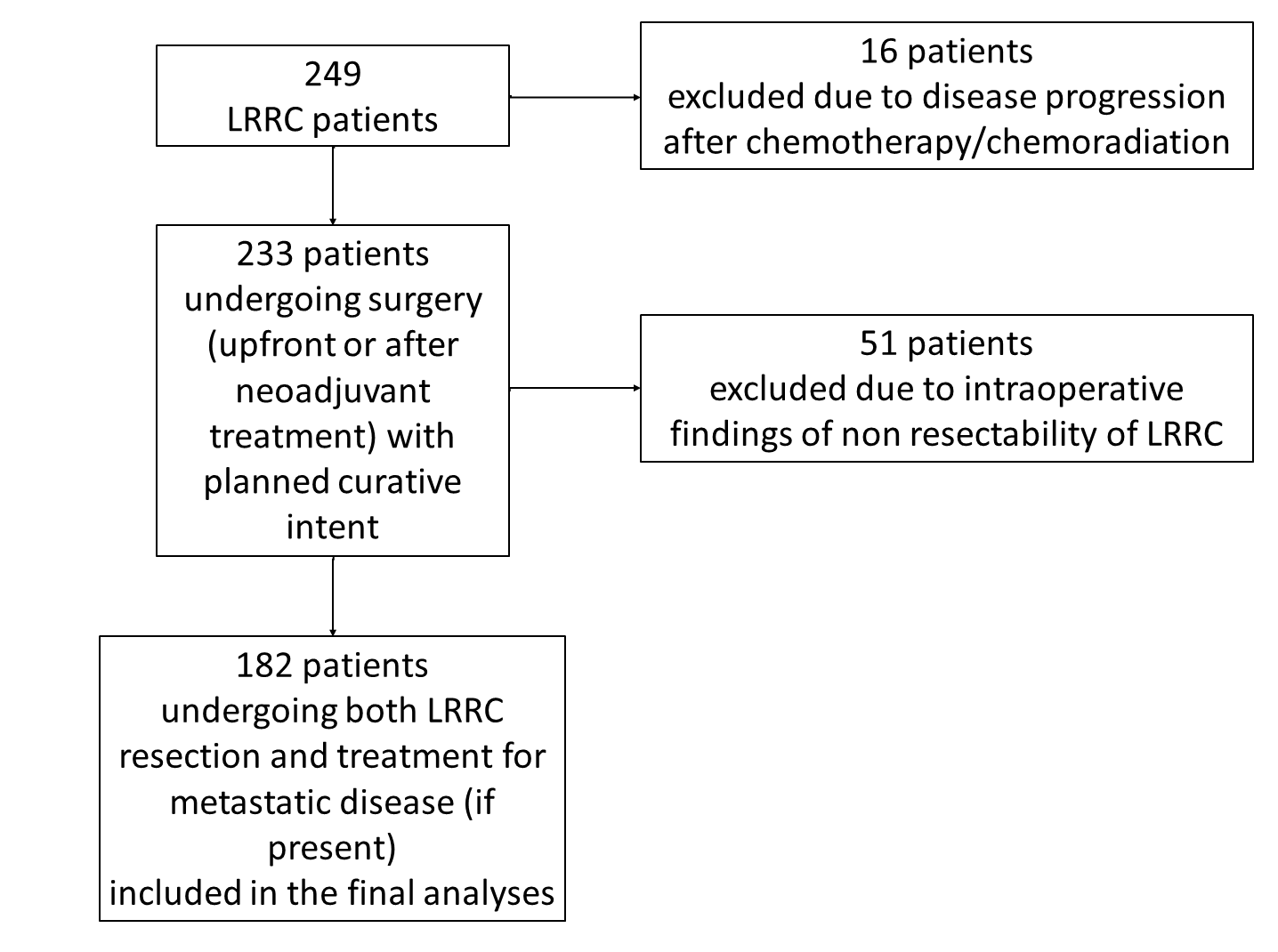
**

**Supplementary Figure S2** Next-generation sequencing panel of 14 patients affected by locally recurrent rectal cancer included in the study


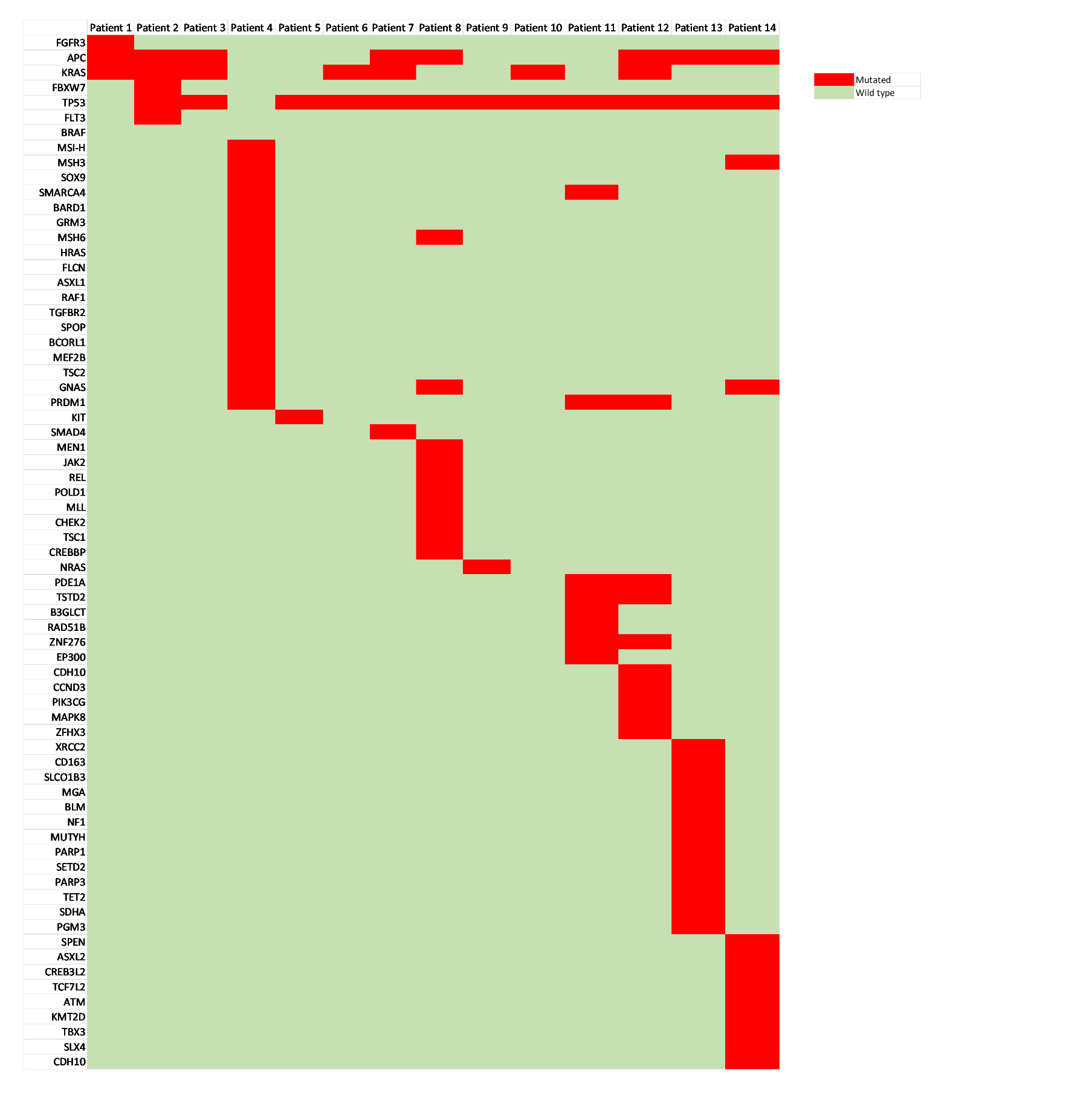


**Supplementary Figure S3** Re-local recurrence-free (a) and distant progression-free survivals (b) in patients affected by previous metastases with primary rectal cancer, locally recurrent rectal cancer with and without concurrent distant metastases; re-local recurrence-free (c) and distant progression-free survivals (d) specifically in patients who completed the planned multimodal treatment with curative intent


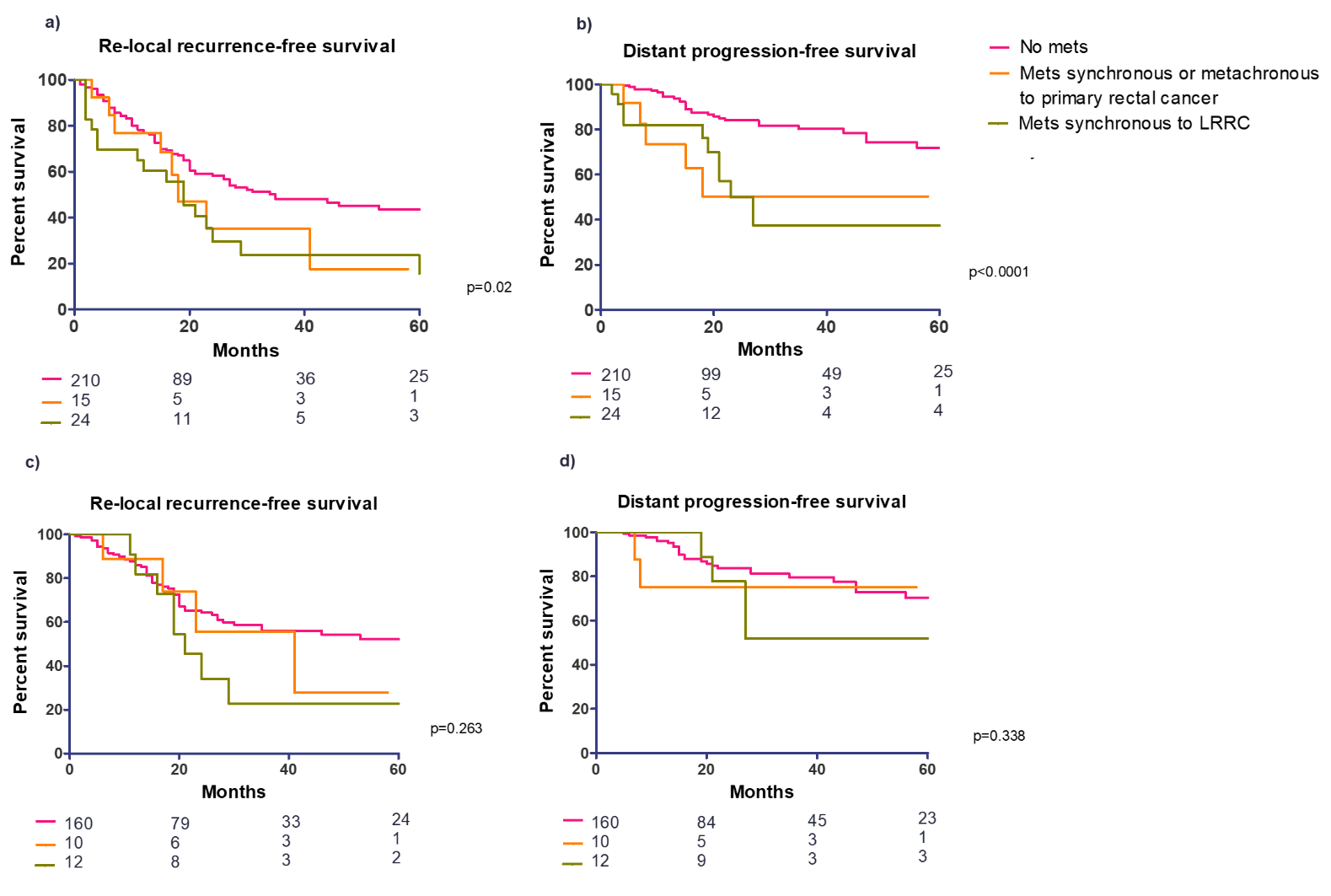

Supplement: zrae061_Supplementary_Data [file zrae061_supplementary_data.docx]
